# Hidden within a pandemic: how is international funding supporting mental health during COVID-19?

**DOI:** 10.1017/gmh.2022.19

**Published:** 2022-03-14

**Authors:** Rebecca S. F. Gribble, Jenna R. Durham, Samantha F. Roy

**Affiliations:** Georgetown University, Washington, DC, USA

**Keywords:** COVID-19, international financing, mental health

## Abstract

**Background:**

The coronavirus disease 2019 (COVID-19) pandemic is bringing to light the long-neglected area of mental health. Current evidence demonstrates an increase in mental, neurological and substance use conditions globally. Although long-established as a leading cause of disease burden, mental health has been historically grossly underfunded. This analysis seeks to demonstrate the extent to which funding for mental health has been prioritised within the international COVID-19 response.

**Methods:**

The authors analysed the development and humanitarian funding through data provided by the International Aid Transparency Initiative. Project-level COVID-19 data from January 2020 to March 2021 were reviewed for mental health relevance. Relevant projects were then classified into categories based on populations of concern for mental health and the degree of COVID-19 involvement. Financial information was assessed through project transaction data in US Dollars.

**Results:**

Of the 8319 projects provided, 417 were mental health relevant. Mental health-relevant funding accounted for less than 2% of all COVID-19 development and humanitarian funding. Target populations which received the majority of mental health relevant funding were children and humanitarian populations, and 46% of funding went towards activities which combined COVID-19 responses with general humanitarian actions. Over half of mental health relevant funding was received by ten countries, and ten donor organisations provided almost 90% of funding.

**Conclusion:**

This analysis shows that the international donor community is currently falling short in supporting mental health within and beyond the COVID-19 pandemic. As the pandemic continues, sustainable country-led awareness, treatment, and prevention for mental, neurological and substance use conditions must be prioritised

## Introduction

Mental, neurological, and substance use (MNS) conditions have long attracted attention on the global health stage for their high contribution to the global disease burden but are yet to receive sufficient financial support in health and development responses. In 2019, MNS conditions accounted for over 10% of the global burden of disease (in disability-adjusted life years), and almost a quarter of all years lived with disability (Global Burden of Disease Collaborator Network, 2019). Yet in the decade preceding this, just 0.3% of development assistance for health was dedicated to mental health (Liese *et al*., 2019).

Globally, mental health has historically been underfunded. Prior to coronavirus disease 2019 (COVID-19), studies estimated development assistance for mental health (DAMH) at less than 1% of all development assistance for health (Gilbert *et al*., 2015; Charleson *et al*., 2017; Turner *et al*., 2017; Lu *et al*., 2018; Liese *et al*., 2019). On average, country spending on mental health amounts to 2% of national health budgets, with little investment in community-based services (UN, 2020). These estimates are the basis for the wide consensus that mental health requires larger funding (UN, 2020; WHO, 2020*a*, 2020*b*). While increased investment in mental health, nationally and globally, was called for prior to the pandemic, particular consideration must now be given to financial investment to areas of greater risk and lower access to services (Kola *et al*., 2021*a*, 2021*b*).

There is a well-established consensus on the detrimental impact of the COVID-19 pandemic on mental health. It is known that individual quarantine has negative psychological effects, as seen prior to the COVID-19 pandemic with the severe acute respiratory syndrome (SARS), influenza, Ebola and Middle East respiratory syndrome (MERS) (Brooks *et al*., 2020). Considering that these prior outbreaks did not have the global reach which COVID-19 quickly obtained, there was early and substantiated concern for mental health impacts. Since the beginning of the COVID-19 pandemic an increase in depressive and anxiety disorders has been observed in global populations, alongside an increase in depressive symptoms in the United States and a decrease in mean population mental health in the United Kingdom (COVID-19 Mental Disorders Collaborators, 2021; Ettman *et al*., 2021; Pierce *et al*., 2021). These outcomes have been associated with a reduction in mobility (lockdowns), increasing infection rates, individual infection with the SARS-CoV-2 virus, and financial difficulties (COVID-19 Mental Disorders Collaborators, 2021; Ettman *et al*., 2021; Pierce *et al*., 2021). Studies in China have shown survivors of COVID-19 to have higher levels of MNS conditions than a general population upon discharge from hospital, including findings which indicate higher rates of depression and anxiety at 12 months compared to 6 months after infection and treatment (Mei *et al*., 2021; Huang *et al*., 2021). In consideration of COVID-19 driven socioeconomic challenges, one forecast estimates a potential increase in excess suicides in the US (up to 3.3–8.4% per year) (McIntyre and Lee, 2020).

Early in the pandemic, the United Nations released a policy brief calling for action on mental health as part of the COVID-19 response and identifying specific populations of concern. These included: first responders and frontline workers; older adults and people with pre-existing health conditions; children; adolescents and young people; women; and people in humanitarian and conflict settings (United Nations, 2020). While much of the initial focus on the mental health implications of the COVID-19 pandemic was towards high-income countries, it is critical to acknowledge that many high-risk populations for MNS conditions are living in low- or middle-income countries, which have historically received low amounts of international funding for mental health (Kola *et al*., 2021*a*).

Previous estimates of DAMH can be further explored in regard to the populations of concern for mental health listed above. It is estimated that one in five people affected by conflict will have an MNS condition, however, the humanitarian and emergency response sectors receive approximately 20% of DAMH (roughly corresponding to 0.2% of all development assistance for health) (Gilbert *et al*., 2015; Liese *et al*., 2019; WHO, 2019). Additionally, it is estimated that children and adolescent populations receive 12.5–16% of all DAMH, despite MNS conditions being a leading cause of overall disability within this group (Lu *et al*., 2018; Turner *et al*., 2017; Global Burden of Disease Collaborator Network, 2019).

Given the historical underfunding of mental health in development assistance and current increased need, the question of financial support for mental health within international COVID-19 responses clearly calls for assessment. In March 2020, the United Nations launched the COVID-19 Global Humanitarian Response Plan, a $10.3 billion appeal to protect the world's most vulnerable from COVID-19, which included the increased need for mental health and psychosocial support (UNOCHA, 2020*a*). At its end in December 2020, just 40% of the appeal had been funded (International Rescue Committee & Development Initiatives, 2021).

This paper seeks to analyse the development and humanitarian funding for mental health within international COVID-19 funding. Using activity-level data from the International Aid Transparency Initiative (IATI), this paper demonstrates the consideration mental health has been given in international COVID-19 responses. Using the populations of concern identified by the United Nations, we present an initial picture of how funding is supporting MNS conditions in the context of the ongoing pandemic.

This is a timely assessment of international funding for mental health, in response to a pressing disease burden as well as the prominence mental health continues to gain on the international health agenda. In May 2021 the World Health Assembly approved the updated World Health Organization Comprehensive Mental Health Action Plan (2021–2030) which included mental health during emergencies for the first time (WHO, 2021*a*). This is a clear indication of the importance mental health plays in ensuring health and wellbeing during and after the COVID-19 pandemic, and in meeting the 2030 Sustainable Development Goals.

## Methods

Development and humanitarian funding for mental health within COVID-19 funding were investigated using data from the IATI. The IATI was chosen as a data source for several reasons. First, the IATI's database makes it possible to review humanitarian and development funding globally in a manner that is standardised and includes various indicators per activity such as programme aims, location, and financial transaction. IATI receives reporting from donors across the governmental, non-governmental, and private sectors (philanthropy), allowing for a comprehensive review of multiple funding sources (IATI, 2022*a*). Second, in an effort to support the global response to the COVID-19 pandemic, IATI developed specific guidance for organisations to publish data on their international development and humanitarian activities responding to COVID-19 (IATI, 2020). This platform enables information sharing and provides data which is open and free with the aim to improve the ‘transparency of development and humanitarian resources and their results for addressing poverty and crises’ (IATI, 2022*b*). At the time of data collection for this paper, Organisation for Economic Co-operation and Development (OECD) data – which provides similar data elements – was not yet available for the COVID-19 timeframe.

Activity-level data from the IATI Registry was provided to the authors based on being classified as COVID-19 funding: determined by having been flagged and coded as COVID-19 relevant by reporting organisations, the project being assigned a relevant humanitarian scope code by IATI or the reporting agency, or having ‘covid’ present in an activity description (A. Silcock [IATI], personal communication, 25 February 2021). A total of 8319 activities that were designated as COVID-19 funding by IATI classification were received by the authors. All activities provided by IATI were analysed for mental health relevance through a keyword search. The keywords used to identify activities which involved mental health are terms which have been previously established to identify and analyse DAMH (Gilbert *et al*., 2015; Liese *et al*., 2019). Please see online Supplementary Table S1 for a full list of search terms. Due to the basic function of this process, activities with any variation or usage of a keyword were identified. To ensure mental health relevance, all activities were manually reviewed for relevance and classification.

Once selected for inclusion, activities relevant to mental health were classified into categories based on target recipient population and COVID-19 response. The descriptive data elements of ‘project title’, ‘title narrative’, ‘description narrative’, and ‘participating organisation narrative’ were used to review and classify activities. Target populations were the specific populations of concern as identified in the United Nations Policy Brief: COVID-19 and the Need for Action on Mental Health: first responders and frontline workers, older adults and people with pre-existing health conditions, children, adolescents and young people, women, and people in humanitarian and conflict settings (United Nations, 2020). The authors added two additional target populations: those directly affected by COVID −19 (diagnosed with COVID-19, caring for individuals diagnosed with COVID-19, or under personal public health COVID-19 interventions such as quarantine); and populations with pre-existing MNS conditions. These populations were included to further reflect the growing relationship between COVID-19 and mental health and to account for the already high burden of MNS conditions. The target population most directly connected to the mental health element of the activity was identified as the primary target population, secondary target populations were recorded when additional and/or broader populations were also included in the activity description. While all activities provided to the authors were classified as COVID-19 funding by IATI, the mental health-related elements of these activities were found to have varying degrees of COVID-19 response. To better represent the link between mental health and COVID-19, activities were also reviewed to assess the degree of COVID-19 response in regard to the mental health element of the activity (for example, was the mental health element in direct response to COVID-19 quarantine or was it in response to general humanitarian circumstances). The mental health elements of activities were classified into one of the following groups: a direct response to COVID-19, a combination of COVID-19 and general development assistance, development assistance in the time of COVID-19, and no COVID-19 response for the mental health element of the activity. Please see online Supplementary Table S2 for examples of activity classification.

All activities which were identified as mental health relevant and categorised based on target populations and COVID-19 response were then assessed for their financial information. Activity identification numbers were sent to IATI, who then provided all associated transactions. Transaction data was reviewed by transaction type, with disbursements, reimbursements, and expenditures included for analysis (removing commitments). Values for each transaction were provided in US Dollars. Transactions are converted into US Dollars by IATI from the currency of publication on the date of transaction (for this analysis, these dates occurred between January 1st 2020 and March 31st 2021). Analysis was performed using Microsoft Excel. If activity information was provided in a language other than English, Google Translate was used.

## Results

From the 8319 activities provided by IATI, the mental health keyword search returned 797 activities which included any of the search terms used. Upon assessment of appropriateness for inclusion a significant number of activities obtained by the keyword search were excluded due to their objectives not being pertinent to mental health (for example, ‘depression’ used in the weather-related term ‘tropical depression’). This resulted in 417 activities relevant to mental health available for analysis. As described in the methods section, activities were classified based on their target recipient population(s) and the degree of COVID-19 response within the mental health element of the activities objectives.

The 417 activities relevant to mental health returned 1625 transactions from IATI, with a total value of US$979868798.20. Some (37) activities did not have transaction data available and were therefore not included further in the analysis. The transactions obtained from IATI for the relevant activities included transactions that were dispersed between 2017 and 2021. The United Nations Office for the Coordination of Humanitarian Affairs tracks COVID-19 funding from January 1st 2020 onwards (UNOCHA, 2021). Therefore, only transactions occurring after January 1st 2020 were included in this analysis to maintain consistency and clarity in international COVID-19 funding. Transactions which occurred prior to January 1st 2020 were for activities that began before the pandemic and had been repurposed for COVID-19 at a later date. While these activities are ongoing, and were adapted to the COVID-19 environment, transactions which occurred prior to this time were not intended as a COVID-19 response (however, transactions for these activities which occurred after January 1st 2020 were included). The total value of all activities relevant to mental health in response to COVID-19, from January 2020 to March 2021, was US$871636741.10.

The total value of all 8319 activities provided by IATI as COVID-19 funding was also obtained. Transactions for these activities were analysed from January 1st 2020 to March 31st 2021. Total development and humanitarian funding for COVID-19, per IATI data provided, was US$46.9 billion for this time period. Mental health-relevant funding then accounts for less than 2% of all COVID-19 development and humanitarian funding analysed.

While the US$871.6 million in value of activities relevant to mental health within COVID-19 funding is used for analysis in this paper, this amount represents a gross overestimation of international support for mental health. Activities which were identified through the keyword search contain an element of mental health care, prevention, or awareness but these frequently occur alongside other non-mental health-related activity aims. For example, an activity may include psychosocial counselling alongside the provision of general medical care and humanitarian responses such as shelter and education. Activities entered within the IATI database do not currently include cost breakdowns between elements, meaning that it is not possible to separate out spending for mental health-related elements within these activities at this point in time (IATI, 2020). This data characteristic has also been noted when analysing DAMH with OECD data (Liese *et al*., 2019). Therefore, the portion of funding dedicated to mental health within these activities is unknown. For the purpose of this paper, the authors referred to activities as ‘relevant to mental health’ which serves as an indication of mental health consideration within the international COVID-19 response.

Within this total value of US$871.6 million, 63 countries were recipients of activities relevant to mental health within the international COVID-19 response. However, just ten countries received over half (66.5%) of mental health relevant funding, with the top 20 recipient countries receiving over 88.4% of all funding. The top recipient countries were Turkey (16.4%), Iraq (11.9%), Democratic Republic of the Congo (6.2%), Lebanon (6.2%), Mali (4.6%), Sudan (4.6%), South Sudan (4.5%), Libya (4.3%), Bangladesh (4.0%), and Haiti (3.9%).

There were 61 governmental, non-governmental, and private donors which provided development and humanitarian funding relevant to mental health as identified by this paper. Of these, just ten contributed the vast majority (89.6%). These donors were the German Federal Ministry for Economic Cooperation and Development (31.4%), the United Nations Office for the Coordination of Humanitarian Affairs' Central Emergency Response Fund (21.4%), the Canadian Department of Foreign Affairs, Trade and Development (17.3%), the European Commission's European Civil Protection and Humanitarian Aid Operations (7.2%), Sweden (2.8%), The United States of America's United States Agency for International Development (2.4%), Syria Cross-border Humanitarian Fund (2.5%), Netherlands Ministry of Foreign Affairs (1.9%), the Democratic Republic of the Congo Humanitarian Fund (1.7%) and the Ethiopia Humanitarian Fund (1.3%). The Syria Cross-border Humanitarian Fund, the Democratic Republic of the Congo Humanitarian Fund, and the Ethiopia Humanitarian Fund are multi-donor country-based pooled funding mechanisms to support each country's humanitarian response plan. The United Nations Office for the Coordination of Humanitarian Affairs' Central Emergency Response Fund is the United Nations' global emergency response fund which also pools contributions from donors into a single fund (UNOCHA, 2020*b*, n.d. *a*, n.d. *b*).

**Fig. 1.**
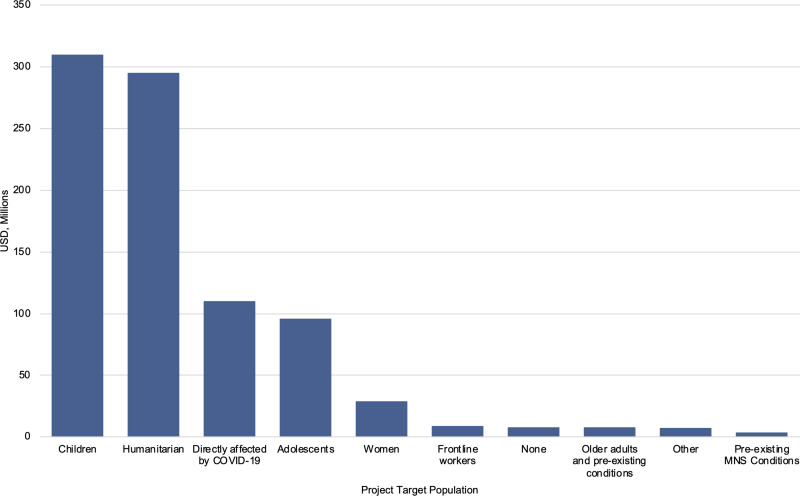
Populations of concern as recipients of mental health funding within COVID-19 funding, in USD millions.

Of the US$871.6 million in funding for activities which were relevant to mental health in response to COVID-19 globally, the majority went towards two specific populations of concern: children and humanitarian populations (35.5% and 33.8% respectively). As seen in Fig. 1, populations directly affected by COVID-19, were the third-largest population of concern to receive funding (12.6%). Women, first responders and front line workers, older adults and those with pre-existing health conditions, and those with pre-existing MNS conditions are all populations which received very small amounts of funding for mental health within COVID-19 responses (3.3, 1.0, 0.9, and 0.4% respectively).

In regard to COVID-19 response, mental health relevant funding was predominantly directed towards activities which combined COVID-19 with general humanitarian responses (46.0% of the US$871.6 million). However, 37.0% of all mental health relevant funding was directed towards activities which were in direct response to COVID-19. The remaining funding was directed towards general humanitarian aid delivery in the time of COVID-19 (10.0%) and towards activities where the mental health element had no stated relevance to COVID-19 (7.0%).

**Fig. 2.**
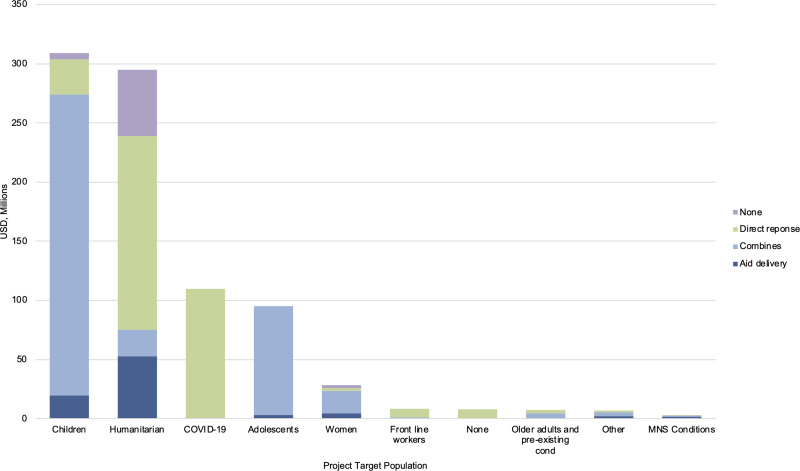
Target population of concern and COVID-19 response for mental health funding within COVID-19 funding, in USD millions.

In Fig. 2, mental health relevant funding is represented by the specific target population and COVID-19 response within these populations. Of note is the funding directed towards populations directly affected by COVID-19 and frontline workers were in direct response to the COVID-19 pandemic. Populations with pre-existing MNS conditions however, received the majority of funding through aid delivery in the time of COVID-19 (i.e. ongoing aid delivery which recognises, and works within pandemic guidance, but does not change the activity objectives in regard to COVID-19). Women, adolescents, children, and older adults and those with pre-existing health conditions mainly received funding through combined COVID-19 and general humanitarian responses. General humanitarian populations received the largest amount of mental health relevant funding in direct response to COVID-19.

**Fig. 3.**
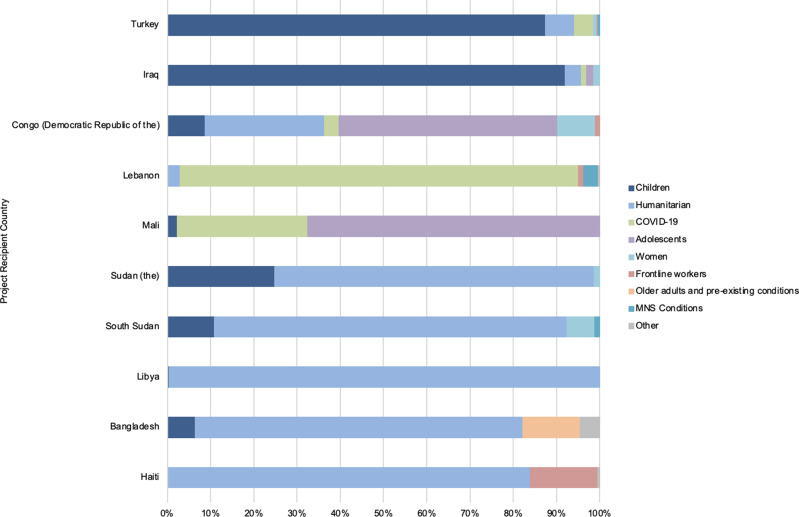
Top 10 activity recipient counties with populations of concern as recipients of mental health funding within COVID-19 funding (%).

The specific populations of concern for mental health during the COVID-19 pandemic were also examined within the top ten recipient countries, as seen in Fig. 3. The majority of funding in Sudan, South Sudan, Libya, Bangladesh, and Haiti went towards general humanitarian populations. While in Turkey and Iraq the majority of funding went towards children. Lebanon was the only top recipient country where the majority of funding went towards those directly affected by COVID-19. Only South Sudan, Lebanon and Turkey saw funding that went towards populations with existing MNS conditions, and only Bangladesh saw funding that went towards older adults and those with pre-existing health conditions within the top ten recipient countries. Haiti had the largest proportion of funding that went towards first responders and frontline workers within these ten countries.

## Discussion

This assessment of development and humanitarian funding for mental health within the international COVID-19 response shows a continuation of previous funding patterns for mental health as well as some encouraging outcomes. Similar to previous research which places DAMH at no more than 1% of all development assistance for health, this indication places funding *relevant* to mental health at less than 2% of all COVID-19 funding (Gilbert *et al*., 2015; Charlson *et al*., 2017; Liese *et al*., 2019). Considering this is an overestimation of funding for mental health, as mental health was just one element of the activities identified, absolute amounts of funding are likely to be similar to pre-COVID-19 levels. This is despite the recognition of mental health being an essential part of the international COVID-19 response by the international health community, researchers and countries.

Mental health is an important element of COVID-19 response, alongside laboratory facilities, vaccine development and delivery, and acute health care provision, to lessen long-term social and economic costs to society together with a reduction in individual suffering (UN, 2020).

The mental health impacts of the COVID-19 pandemic are predicted to be significant, enduring, and to further exacerbate inequity in global mental health (Kola *et al*., 2021*a*, 2021*b*). Considering the populations who are recipients of humanitarian funding are also those who are historically under-resourced for mental health, there needs to not only be a continuum of funding but an increase in investment for mental health during and post the pandemic.

The majority of countries in an assessment by the World Health Organization had mental health and psychosocial services included in their national COVID-19 response plans, however, less than one-fifth of these countries had full government funding for these activities (WHO, 2020*b*). All low-income countries surveyed had mental health and psychosocial services included in their national COVID-19 response plans, yet the majority had zero national funding allocated for these services (WHO, 2020*b*). Prior to the COVID-19 pandemic, government mental health expenditure as a percentage of general government health expenditure was just 1.05% in low-income countries, 1.10% in lower-middle and 1.60% in upper-middle-income countries (WHO, 2021*b*). With little funding from international donors, and low-levels of investment at the domestic level, many national-level mental health plans are left unfunded and therefore unrealised.

The top recipient countries of mental health relevant funding within COVID-19 funding as identified by this analysis are mainly conflict- and disaster-affected (or receiving nations for conflict- and disaster-affected refugees). These results are similar to previous research on international funding for mental health, with DAMH prior to COVID-19 predominantly going towards conflict-affected countries (Gribble *et al*., 2021). Similarly, previous research has shown the dominance of just a handful of donors in providing mental health funding, including the European Union, Germany, the United States, Norway and Canada, as found in this analysis (Gribble *et al*., 2021). This emphasises the narrow range of resources available to support the large burden of mental health globally. As noted in the results, the majority of the funding came from donor countries and United Nations bodies. Given that IATI reporting is voluntary (although well supported), this data may not fully capture the role of the private philanthropic sector in financing mental health during COVID-19 responses. However, these results show there is room for the private philanthropic sector to contribute to global mental health programming.

Of note in these results is how populations directly affected by COVID-19 were the third-largest group to receive mental health funding within COVID-19 funding. This is an encouraging result, indicating that mental health was considered in the pandemic response. An example of an activity element demonstrating this is: ‘*As it* [*is*] *now well known that the contraction of the Covid-19 disease and the isolation or quarantine period has a strong impact on the **mental health** and well-being of persons, **psychosocial** support will be implemented to patients and families, with a specific focus on vulnerable groups as children, pregnant women, elderly* [*…*] ***MHPSS** topics will be implemented to ensure medical staff are well aware of the disease and its implications’.* At the time of data collection, long-COVID had not yet emerged as a concern, and therefore would not be reflected in this data. Mental health activities for those affected by COVID-19 in this paper are in relation to the immediate effects of the disease. However, this should be a changing consideration going forward.

Some target populations of concern received small amounts of support as the primary recipients of mental health activities. These include women, first responders and frontline workers, older adults and those with pre-existing health conditions and populations with pre-existing MNS conditions. Women received just a fraction of the funding identified by this paper despite resounding warnings regarding an increase in violence against women and girls (particularly domestic/intimate partner violence) since the beginning of the pandemic (United Nations Entity for Gender Equality and the Empowerment of Women, 2020). Intimate partner violence is associated with an increase in MNS conditions such as depression, post-traumatic stress disorder and suicide attempts alongside physical injuries (World Health Organization, 2021*c*). When looking deeper within the primary target group of humanitarian populations, women were the secondary target population for 46% of mental health funding. While secondary populations are not the primary (or sole) recipients of support through these activities, it is salient that women are being recognised in funding for mental health during COVID-19.

Similarly, first responders and frontline workers received very limited funding as identified by this paper. Yet looking at funding for those directly affected by COVID-19, 51% of this went towards the secondary population of first responders and frontline workers. There are clear indications of the mental health burden which responding to COVID-19 places on first responders and frontline workers, with one report finding 62% of healthcare frontline workers stating that pandemic-related stress has negatively affected their mental health, and reports of COVID-19 frontline mental health counsellors needing counselling themselves (Anderson, 2021; Kaiser Family Foundation, 2021).

The two other populations of concern which received the smallest amounts of funding were older adults and those with pre-existing conditions and populations with pre-existing mental health conditions. While older adults and those with pre-existing conditions received very small amounts of funding, there were a handful of projects which directly identified mental health concerns in this population, an example of this is: ‘*Strengthening the capacity of older people structures in order to address the information gaps on COVID-19; promote referral linkages of suspected cases; and address isolation, **psychosocial trauma** and fear among older people. To strengthen national health COVID-19 responsiveness in delivering COVID-19 services, guidance and advice for older people with NCDs and other conditions*’. This shows a clear recognition of needing to provide services appropriate for this population to reduce distress among a handful of donors. Given the historical underfunding of mental health in development assistance prior to COVID-19, the results for populations with pre-existing mental health conditions are not surprising. These four findings emphasise the need to prioritise populations of concern within increased support for mental health awareness, treatment, and prevention globally.

This paper is intended as a timely indication of development and humanitarian funding for mental health within the international COVID-19 response. This aligns both with calls (and now evidence) for increased support for mental health as well as aligning with growing demands for transparency within international health funding initiatives (Global Burden of Disease 2020 Health Financing Collaborator Network, 2021; International Rescue Committee & Development Initiatives, 2021).

There are limiting factors for using data from IATI. IATI encourages all organisations who contribute resources to developing countries to report their activities. While to date there are over 1350 governments, multilateral institutions, private sector, and civil society organisations who publish data with IATI, this does not reflect all active international donors or capture all activities in the international donor community. However, the IATI standard for reporting, and the resultant data, is considered a benchmark for transparency of development and humanitarian resources. Another limitation of using IATI data is the potential for double-counting activities and therefore funding. As IATI receives information from multiple sources, the same funding may be reported multiple times as it flows between different organisations. The authors of this paper examined all activities provided to look for potential funding which may be double-counted by looking at organisations which were listed as both recipients and donors. However, no activities were able to be removed from the analysis in regard to double-counting due to all activity information being different between incoming and outgoing funding from these organisations.

Further limiting factors of this paper were the use of English-only search terms and the variation in the amount of information provided for each project; some activity descriptions were very detailed while others were just a few words. This means the authors made assumptions regarding the inclusion and classification of activities which may have affected the final outcomes of the analysis. Furthermore, the time taken to manually review project-level data may limit the ability of this paper to be reproduced or for the analysis to be applied over a longer period of time.

This analysis serves as an initial indication of development and humanitarian funding for mental health within the international COVID-19 response. Given the increasing attention that MNS conditions are receiving during the ongoing COVID-19 pandemic, yet the low levels of funding, this paper may act as a baseline for awareness, discussion and improvement of current and future support for mental health.

## Conclusion

Mental health is an essential consideration for responding to the global COVID-19 pandemic, as emphasised by countries, international organisations, and evidenced by emerging research. Just US$871.6 million in COVID-19 humanitarian and development assistance was found to be supporting activities which involve an element of mental health, among other elements, during the first year of the pandemic. Funding for mental health is currently falling short in supporting populations of concern within - and beyond - the COVID-19 pandemic. As the pandemic continues, there is scope to improve sustainable country-led awareness, treatment and prevention for mental, neurological and substance use conditions.

## Supplementary material

For supplementary material accompanying this paper visit https://doi.org/10.1017/gmh.2022.19. click here to view supplementary material
