# Supplementary material for: Hidden within a pandemic: how is international funding supporting mental health during COVID-19?
Source: Glob Ment Health (Camb). 2022 Mar 14;9:173–80. doi: 10.1017/gmh.2022.19 (PMC8961071; doi:10.1017/gmh.2022.19)
Supplement: Supplementary file 1 [file S205442512200019Xsup001.docx]

**Supplemental Tables:**

Table 1: Mental Health-Related Keyword Search Terms and Results.

| **Keyword** | **Source / New Term** | **Results (No. Projects containing this term)** **from initial keyword search (before assessing mental health relevance)** |
| --- | --- | --- |
| Addiction | Liese et. al. 2019 | 1 |
| ADHD | New terminology added | 0 |
| Affective | Liese et. al. 2019 | 0 |
| Alcohol | Liese et. al. 2019 | 75 |
| Antidepressant | Liese et. al. 2019 | 0 |
| Antipsychotic | Liese et. al. 2019 | 0 |
| Anxiety | Liese et. al. 2019 | 7 |
| Anxiolytic | Liese et. al. 2019 | 0 |
| Attention Deficit | New terminology added | 0 |
| Autism | Liese et. al. 2019 | 0 |
| Behavior therapy/behaviour therapy | Liese et. al. 2019 | 1 |
| Bipolar | Liese et. al. 2019 | 0 |
| Cognitive | Liese et. al. 2019 | 9 |
| Coping | New term added based on data terminology | 96 |
| Counseling | New term added based on data terminology | 32 |
| Delirium | Liese et. al. 2019 | 0 |
| Delusion | Liese et. al. 2019 | 0 |
| Dementia | Liese et. al. 2019 | 0 |
| Dependency | Liese et. al. 2019 | 8 |
| Depressi^1^ | Liese et. al. 2019 | 4 |
| Developmental | Liese et. al. 2019 | 13 |
| Downs syndrome | Liese et. al. 2019 | 0 |
| Drug use | New term added based on data terminology | 2 |
| Eating disorder | Liese et. al. 2019 | 0 |
| Electroconvulsive therapy | Liese et. al. 2019 | 0 |
| Emotion | New term added based on data terminology | 12 |
| Epilep^2^ | Liese et. al. 2019 | 2 |
| Hallucination | Liese et. al. 2019 | 0 |
| Hyperactivity | Liese et. al. 2019 | 0 |
| Hypochondriasis | Liese et. al. 2019 | 0 |
| Insomnia | Liese et. al. 2019 | 0 |
| Learning disability | Liese et. al. 2019 | 0 |
| Mania | Liese et. al. 2019 | 0 |
| Mental | Liese et. al. 2019 | 54 |
| Mental health | New term added based on data terminology | 27 |
| MHPSS | New term added based on data terminology. Abbreviation for mental health and psychosocial services. | 24 |
| MH | New term added based on data terminology. Abbreviation for mental health. | 0 |
| Mental wellbeing/mental well-being | New term added based on data terminology | 6 |
| Mood | Liese et. al. 2019 | 0 |
| Neuro^3^ | Liese et. al. 2019 | 3 |
| Personally disorder | Liese et. al. 2019 | 0 |
| PFA | New term added based on data terminology. Abbreviation for psychological first aid. | 0 |
| Phobia | Liese et. al. 2019 | 0 |
| PSS | New term added based on data terminology. Abbreviation for psychosocial services. | 18 |
| Psych^4^ | Liese et. al. 2019 | 316 |
| PTSD | New terminology added | 0 |
| Schizophrenia / schizophrenic | Liese et. al. 2019 | 0 |
| Self harm/self-harm | Liese et. al. 2019 | 0 |
| self-help/self help | New term added based on data terminology | 13 |
| Sleep disorder | Liese et. al. 2019 | 0 |
| Social isolation | New term added based on data terminology | 0 |
| Socioemotional | New term added based on data terminology | 0 |
| Somatoform | Liese et. al. 2019 | 0 |
| Stress | Liese et. al. 2019 | 39 |
| Substance abuse | Liese et. al. 2019 | 0 |
| Substance use | Liese et. al. 2019 | 0 |
| Suicide | Liese et. al. 2019 | 0 |
| Trauma^5^ | Liese et. al. 2019 | 35 |
| *Please note: To avoid duplication, once a project was found to contain a keyword it was excluded from further keyword searches. (i.e. if a project contains both the terms “mental health” then “psych” it will only be counted once as a result for “mental health”).*  *^1^Depressi was used to capture both ‘depression’ and ‘depressive’*  *^2^Epilep was used to capture both ‘epilepsy’ and ‘epileptic’*  *^3^Neuro was used to capture both ‘neurologic’ and ‘neurological’*  *^4^Psych was used to capture ‘psychiatry’, ‘psychiatric’, ‘psychological’, ‘psychotic’, ‘psychosis’, ‘psychoses’, and ‘psychosocial’.*  *^5^Trauma was used to capture ‘trauma’ and ‘traumatic’ (this would include post traumatic stress disorder)* | | |

Table 2: Example Activity Classifications for Mental Health Relevance, Target Population, and COVID-19 response.

| **Category** | **Example from data** | | |
| --- | --- | --- | --- |
| Eliminated: use of keyword not applicable to mental health | *“In October 2020, torrential rain caused by a tropical convergence zone combined with cold air mass (05 Oct), tropical storms no. 6 Linfa and no. 7 Nangka, additional tropical* ***depression*** *resulted in severe floods in all the 16 main river systems in the Central coastal area of Vietnam…”* | | |
| **Category** | **Example from data** | **Target population(s)** | **COVID-19 response** |
| Retained: mental health relevant | *“…Improved* ***mental health*** *and* ***psycho socia****l well being of caregivers and children in Ghor province in response to conflict, disaster and COVID-19…”* | *Primary:* Children  *Secondary:* Humanitarian | Combined |
| Retained: mental health relevant | *“…****Mental health*** *and* ***psycho-social*** *support to children and families and provision of water and sanitation facilities for the most in need schools as part of the Save the Children's COVID response...”* | *Primary:* Children  *Secondary:* Other | Direct response |
| Retained: mental health relevant | *“…Cordaid targets beneficiaries who survives through GBV, child protection, Provision of proper and safe places through rehabilitation of educational, health facilities including WASH facilities, the  Activities including* ***MHPSS****, social and life skills to promote non-violent communication, conflict resolution, self reliance and respect and activities such as games sports etc., Cordaid will continue as the member of the consortium for the Syria Joint Response 2020, the organisation will provide activities in the sectors of WASH, heath rehabilitation ,and protection (incl use of cash vouchers) through partners. The focus areas will be Aleppo and rural Damascus…”* | *Primary:* Children  *Secondary:* Humanitarian | None |
| Retained: mental health relevant | *“…The project aims at enhancing the relocation services provided by Tbilisi and Batumi Shelter Cities and Telavi art-residence to the human rights defenders from Central Asia, Russia, Ukraine, Armenia, Belarus, Poland, Hungary, Serbia, Bosnia and Herzegovina and Greece. The Shelter provides HRDs with safe space for work and recovery, offering conditions for physical and* ***psychological*** *rehabilitation, helping to strengthen capacity of human rights defenders and developing the support network for HRDs through organization of seminars, workshops, and meetings. This project is aimed at improving and widening capacity building program for relocated HRDs, strengthening services related to* ***psychological*** *support (collective workshops and individual work with* ***psychologist****) and improving overall conditions in the all shelter facilities and adapting it to the COVID pandemic. The project also contributes to the international advocacy effort for better protection of HRDs. In particular, the project suggests focusing on* ***mental health*** *and freedom of movement of HRDs…”* | *Primary:* Other | Aid delivery in the time of COVID-19 |
